# Efficacy of Cerebrolysin in the reduction of spasticity during stroke rehabilitation

**Published:** 2017

**Authors:** RM Martinez

**Affiliations:** * Department of Rehabilitation Medicine, Amang Rodriguez Memorial Medical Center (ARMMC), Marikina, Philippines

**Keywords:** Cerebrolysin, spasticity, stroke, rehabilitation

## Abstract

Aim: This study assessed the efficacy of Cerebrolysin on post-stroke spasticity, motor recovery, and global functions in an outpatient rehabilitation setting.

Methods: In this retrospective comparison study, Cerebrolysin was administered at a daily dosage of 10 ml for over 30 days as an intramuscular injection. Control patients did not receive Cerebrolysin. All the patients participated in a standardized physical and occupational rehabilitation therapy for one month at least two times per week. Efficacy was assessed at day 30 by using the Modified Ashworth Scale (MAS) for spasticity and the Manual Muscle Testing (MMT) for motor recovery. Global function was assessed by the modified Rankin Scale (mRS) at day 30.

Results: A total of 50 patients were eligible for participation according to the inclusion and exclusion criteria. Of these, 23 patients were treated with Cerebrolysin and 27 patients represented the control group. No significant group differences were observed at baseline. Patients treated with Cerebrolysin experienced a significant reduction of spasticity in muscles of the upper and lower limbs, whereas only minor changes were observed in the control group. In the Cerebrolysin group, differences were statistically significant at day 30. Significant improvements of muscle strength and global functions were observed in both groups at day 30. Cerebrolysin was safe and well tolerated.

Conclusion: Cerebrolysin had a beneficial effect on post-stroke spasticity in an outpatient rehabilitation setting; intramuscular treatment for over 30 days was safe and well tolerated.

## Introduction

Stroke is the second leading cause of mortality and ranks as the fifth leading cause of morbidity in the Philippines [**1**]. The total Filipino population had presently reached the 100 million mark, with a stroke prevalence of 0.9%, of which 70% were of ischemic and 30% of hemorrhagic origin [**2**]. The prevention and treatment of stroke cannot be overemphasized and requires education, awareness, and state of the art interventions. In 2012, the Philippine Academy Rehabilitation Medicine has contextualized guidelines in stroke prevention, treatment, and rehabilitation, which involved the process of translating best-evidence recommendations from good quality guidelines into achievable programs [**3**]. The present advancements in the treatment of stroke have decreased the mortality rate, however, a focus on the co-morbidities affecting body functions, the level of activity and the participation to society is imperative to stroke rehabilitation success. Still, despite the availability of post-stroke rehabilitation, only 54.1% of the stroke survivors are referred to rehabilitation [**4**]. The medical treatment strategies have been made available in the Philippines [**5**,**17**-**23**], but the majority of post-stroke patients experiencing spasticity do not receive treatment and appropriate management, which makes their functional goals in rehabilitation unattainable [**24**].

Previous research on Cerebrolysin was mainly performed in an acute stroke setting with daily drug administration by intravenous infusion, usually of 30 ml for 10 days [**6**-**8**]. Recent randomized, controlled studies have been performed in stroke rehabilitation with a treatment duration of 21 days and concomitant participation in a rehabilitation program [**51**]. These studies reported significant treatment effects of Cerebrolysin on recovery of motor functions in the upper limbs, especially in more severe stroke patients and stroke patients with severe motor impairment. However, no clinical trials have yet investigated the effects of Cerebrolysin on spasticity although inhibitory effects have been reported from experimental studies [**29**-**31**]. 

## Methods

**Study design and treatment regimen**

For this retrospective, controlled, monocentric study, patient records from January to December 2015 were screened for eligible patients. This study investigated the effects of 10 ml Cerebrolysin on the reduction of spasticity in post-stroke patients in an outpatient rehabilitation setting. Patients also participated in a standard rehabilitation program for at least two times per week for one month, which included physical and occupational therapy. This program and treatment with Cerebrolysin were started 4±3 months post-stroke. Effects were compared with patients who also participated in the rehabilitation program but who did not receive Cerebrolysin treatment. Cerebrolysin was administered by intramuscular (IM) injection for over 5 minutes into the deltoid, quadriceps, or gluteus maximus once daily for 30 days. A cold compress was placed on the injection site for 15 minutes. 

**Inclusion and exclusion criteria**

Male and female patients above 18 years old, with ischemic or hemorrhagic stroke, confirmed by computed tomography or magnetic resonance imaging and stable vital signs, were included. Patients did not have a history of seizures and did not receive anti-spasticity or neurotrophic medications during Cerebrolysin therapy. An informed written consent was mandatory for the participation in the rehabilitation program. 

Patients were excluded if hemiparesis or weakness was due to causes other than the ones related to stroke or if they failed to participate in physical or occupational therapy for at least two times per week.

**Efficacy criteria**

Efficacy assessment was based on the Modified Ashworth Scale (MAS) assessing spasticity, the Manual Muscle Testing (MMT) assessing motor recovery and the modified Rankin Scale (mRS) assessing global function; the study endpoint being at day 30. The MAS measures spasticity by assessing resistance during passive soft-tissue stretching [**52**]. The score ranges from 0 (normal tone) to 4 (rigid in flexion or extension). The muscles of the upper extremities assessed according to the MAS by this protocol included the Musculus pectoralis major (pectoralis), the Musculus biceps brachii (biceps), the Musculus flexor carpi radialis (wrist flexor), and the Musculus flexordigitorum profundus (finger flexor). Muscles of the lower extremities assessed by the MAS included the hamstrings, *Musculus tibialis posterior* and *Musculus gastrocnemius*. 

The MMT measures extremity muscle strength but is also used for the assessment of the nervous system in relation to the muscular system [**53**]. The score ranges from 0 to 5; to grade 0-2 the patient is positioned with minimal gravity imposed on the muscle, 3 is described as able to contract the muscle to move the limb against gravity and grades 4-5 is determined by placing resistance to the limb. Muscles assessed according to the MMT by this protocol included the *Musculus biceps brachii* (biceps) and the Musculus quadriceps femoris (quadriceps). [**9**-**13**]

Selection of muscles for the MAS and MMT was done in a pragmatic way, as these are very important muscles for stroke patients in actions such as eating, standing, or walking. 

The mRS measures the degree of disability or dependence in the daily activities from 0 (no symptoms) to 6 (death) [**54**-**56**].

**Safety criteria**

Vital signs were assessed at baseline and at day 30.

**Statistical methods**

Descriptive statistics such as frequency (percentages), mean and standard deviation were computed to describe the demographic and medical profiles of the subjects. Student T-test and Fisher Exact Test were used to compare a significant difference in demographic profiles of the subject between Cerebrolysin and control groups. Paired T-test was computed to determine significant changes in the vital signs (blood pressure, respiratory rates, pulse rates) within groups. Student T-test was calculated to compare vital signs between groups at Day 1 and Day 30. The comparison of spasticity by using MAS, MMT, and Rankin within and between groups, Wilcoxon signed rank test and Mann-Whitney test were performed, respectively. A p-value of less than 0.05 was considered significant in this study. All data were encoded and computed by using SPSS version 23 (IBM Corporation 2015).

## Results

**Study population**

Patient records from January to December 2015 were screened for patients eligible for this study according to the inclusion and exclusion criteria. Of 315 patient records screened, 50 patients were allocated either to the Cerebrolysin (N=23) or control (N=27) group. There were no significant group differences observed at baseline (**Table 1**). The mean age was slightly higher in the Cerebrolysin group (57 vs. 54 years) and male patients were more frequent (Cerebrolysin, 61%; control 88%). Most patients suffered from ischemic than hemorrhagic stroke (Cerebrolysin, 78%, control 81%) and left-sided hemiparesis (Cerebrolysin, 78%; control 52%). Time from stroke to treatment onset was slightly longer in the Cerebrolysin group (4.6 vs. 4.0 months in the controls).

**Table 1:** Demographic baseline characteristics

Parameter	Cerebrolysin, n=23	Control, n=27	
Male sex, n (%)	60.9	77.8	p=0.193
Mean age, y	56.6	54.3	p=0.993
Ischemic stroke, n (%)	78.3	81.5	p=0.777
Right-sided stroke, n (%)	78.3	51.9	p=0.053
Mean time since stroke, months	4.6	5.1	p=0.506

**Efficacy outcome**

**Fig. 1:**
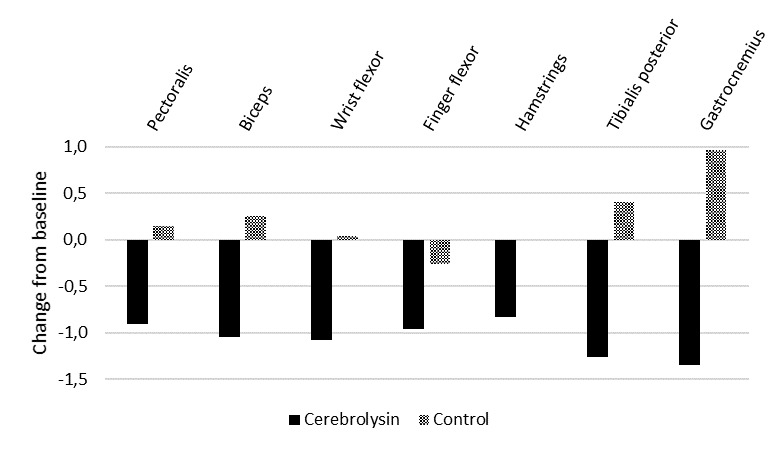
Change from baseline in the Modified Ashworth Scale (MAS) at day 30 measuring spasticity by assessing resistance during passive soft-tissue stretching. Negative score differences indicate improvement. *p<0.05 vs. baseline. Cerebrolysin, n=23; control, n=27

Baseline scores were comparable between study groups in the MAS showing a “more marked” or “considerable” increase in muscle tone. Intragroup comparison showed that patients treated with Cerebrolysin experienced a significant reduction of spasticity in all muscles of the upper and lower limbs as assessed by the MAS at day 30 (**Fig. 1**). Minor changes were observed in control patients showing deterioration in most muscles, which was even significant in the tibialis posterior. These changes resulted in a significant (p<0.05) group difference in favor of Cerebrolysin at day 30 in all muscles assessed (**Table 2**). 

**Table 2:** Scores obtained in the Modified Ashworth Scale (MAS) and Manual Muscle Test (MMT)

	Cerebrolysin			Control			Treatment difference day 30
	Baseline	Day 30	Intragroup difference	Baseline	Day 30	Intragroup difference	
							
Modified Ashworth Scale							
Upper extremity							
Pectoralis	2.39±0.89	1.48±0.67	P=0.002	2.48±0.80	2.63±0.63	P=0.410	P<0.001
Biceps	2.78±0.80	1.74±0.62	P<0.001	2.67±0.55	2.93±0.73	P=0.132	P<0.001
Wrist flexor	2.65±0.93	1.57±0.73	P<0.001	2.52±0.70	2.56±0.75	P=0.868	P<0.001
Finger flexor	2.69±0.88	2.00±0.74	P<0.001	2.78±0.64	2.52±0.85	P=0.167	P=0.022
Lower extremity							
Hamstings	1.61±0.78	0.78±0.80	P<0.001	2.04±0.76	2.04±0.85	P=1.000	P<0.001
Tibialis posterior	2.83±0.83	1.57±0.79	P<0.001	2.74±0.66	3.15±0.53	P=0.016	P<0.001
Gastrocnemius	2.91±0.67	1.57±0.73	P<0.001	2.04±0.75	3.00±0.55	P=0.782	P<0.001
							
Manual Muscle Test							
Upper extremity							
Biceps	1.91±0.85	2.87±0.63	P<0.001	1.59±0.84	2.63±0.56	P<0.001	P=0.175
Lower extremity							
Quadriceps	1.48±0.73	2.70±0.70	P<0.001	1.89±0.85	3.11±0.42	P<0.001	P=0.015

**Fig. 2:**
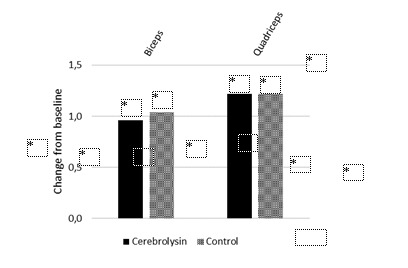
Change from baseline in the Manual Muscle Test (MMT) at day 30 measuring muscle strength by assessing effective performance of a movement in relation to the forces of gravity and manual resistance. Positive score differences indicate improvement. *p<0.05 vs. baseline. Cerebrolysin, n=23; control, n=27

Also in the MMT, baseline scores were comparable between study groups. Intragroup comparisons showed a significant improvement in motor strength of the biceps and quadriceps in both study groups (Table 2). Improvements of muscle strength were similar in both groups (**Fig. 2**). However, at day 30, the strength of the biceps muscle was higher in the Cerebrolysin group but did not reach the level of statistical significance whereas the strength of the quadriceps muscle was in favor of the control group (**Table 2**).

Baseline scores were also comparable for the mRS (Cerebrolysin, 3.7±0.6; control, 3.5±0.6) and patients of both treatment groups improved significantly until day 30. Although patients treated with Cerebrolysin improved to a higher extent (Cerebrolysin, 2.3±0.7; control, 2.6±0.8), treatment differences were not statistically significant at day 30. 

**Safety and tolerability**

Cerebrolysin was well tolerated, no relevant changes were observed in the vital signs, and no adverse events were reported. 

## Discussion

Cerebrolysin is a neuropeptide preparation, which mimics the multimodal action of neurotrophic factors, and was reported to activate A1 adenosine receptors [**29**], alter the paired-pulse facilitation, a presynaptic phenomenon [**25**-**28**], and was unaffected by intracellular application of a potassium channel blocker [**30**]. This study has shown that the administration of Cerebrolysin had a beneficial effect on motor spasticity, which has a prevalence rate of 30% in stroke survivors [**14**,**15**,**16**]. Although modalities and exercises employed in rehabilitation medicine for the treatment of spasticity have been well-documented [**32**-**35**], the control group failed to demonstrate the alleviation of limb spasticity. This might support the proposed inhibitory effect of Cerebrolysin but may also be due to the rather short period of 30 days patients participated in the rehabilitation program. 

Motor recovery from stroke may happen spontaneously by using treatments that limit further brain injury and provide an environment that supports the body to heal itself completely from brain injury. Neuroprotective properties of Cerebrolysin play a vital role here [**36**-**40**]. Furthermore, neuroplastic processes contribute to the recovery process so that stroke survivors with motor deficits will improve performance in activities of daily living despite their limitations. These patients will benefit greatly from the neurorestorative actions of standard rehabilitation and Cerebrolysin [**41**,**42**]. These mechanisms might explain the positive results also seen in control patients regarding better motor recovery and decreasing dependence. 

The neuroprotective and neurorestorative action of Cerebrolysin during the sub-acute and chronic phase of stroke in a rehabilitation setting also enhanced the patients’ motor strength and performance to a higher degree as compared to controls. Cerebrolysin might promote spontaneous healing (3-6 months) and neuroplasticity by collateral sprouting of new synaptic connections and unmasking of previously latent functional pathways (months to years). Additional forms of plasticity include assumption of function by undamaged, redundant neural pathways, reversibility from diaschisis, denervation supersensitivity, and regenerative proximal sprouting of transected neuronal axons shown in various researches in Cerebrolysin [**36**-**42**] and standard rehabilitation treatments [**43**-**50**].

## Conclusion

Cerebrolysin had a beneficial effect on spasticity, improved muscle strength, and global functions in subacute or chronic post-stroke patients in an outpatient rehabilitation setting. Intramuscular administration of Cerebrolysin was safe and well tolerated. Further studies with improved methodology, i.e. randomized controlled trials, appropriate sample size and more reliable, valid, and responsive outcome measures should be considered.
